# An Epidemiological Study of Physical–Mental Multimorbidity in Youth: Une étude épidémiologique de la morbidité physique-mentale chez les jeunes

**DOI:** 10.1177/07067437241271713

**Published:** 2024-08-16

**Authors:** Shannon V. Reaume, Joel A. Dubin, Christopher Perlman, Mark A. Ferro

**Affiliations:** 1 School of Public Health Sciences, 8430 University of Waterloo, Waterloo, Ontario, Canada; 2 Department of Statistics and Actuarial Sciences, 8430 University of Waterloo, Waterloo, Ontario, Canada

**Keywords:** youth, chronic physical illness, mental disorder, multimorbidity, population health, secondary analysis, children and adolescents, disability

## Abstract

**Objective:**

This epidemiological study estimated the lifetime prevalence of chronic physical illness (i.e., an illness that lasted or was expected to last ≥6 months) and 6-month prevalence of mental disorder and multimorbidity (i.e., ≥1 physical illness and ≥1 mental disorder) in youth. Associations between physical illness and mental disorder were quantified, including the number of illnesses. Secondary objectives examined factors associated with mental disorder, after controlling for physical illness.

**Methods:**

Data come from 10,303 youth aged 4–17 years in the 2014 Ontario Child Health Study (OCHS). Physical illness was measured using a list of chronic conditions developed by Statistics Canada. Mental disorders were measured using the OCHS Emotional Behavioural Scales. The Health Utility Index Mark III assessed overall functional health.

**Results:**

Weighted prevalence estimates showed 550,090 (27.8%) youth had physical illness, 291,986 (14.8%) had mental disorder, and 108,435 (5.4%) had multimorbidity. Physical illness was not associated with mental disorder. However, youth with 2 physical illnesses, as compared to no physical illnesses, had increased odds of having any mental (OR = 1.75 [1.08, 2.85]), mood (OR = 2.50 [1.39, 4.48]) and anxiety disorders (OR = 2.40 [1.33, 4.31]). Mean functional health scores demonstrated a dose–response association across health status categories, with the highest scores among healthy youth and the lowest scores among multimorbid youth (all *p *< .05).

**Conclusion:**

Chronic physical illness and mental disorders are prevalent in youth. Youths with 2 physical illnesses have a higher likelihood of mental disorders. Higher functional health scores protected against all mental disorders. Mental health interventions for youth should promote strong overall functional health.

**Plain Language Summary Title:**

Physical-Mental Multimorbidity in Ontario Youth

## Plain Language Summary

This study examined mental disorders in Ontario youth with and without chronic physical illnesses. Youth with at least one physical illness and one mental disorder have physical-mental multimorbidity. 27.8% of youth had chronic physical illness (that lasted at least six months), 14.8% had mental disorder, and 5.4% had multimorbidity. Risk factors for any mental, mood, and anxiety disorders included living with two chronic physical illnesses. Overall functional health of youth declined from youth that were healthy (no physical illness or mental disorder), chronic physical illness only, mental illness only, to multimorbid youth. Youth mental health interventions should promote strong overall functional health.

## Résumé

**Objectifs:**

La présente étude épidémiologique estimait la prévalence de durée de vie de la maladie physique chronique (c'est à dire, une maladie qui durait ou qu'on prévoyait durer ≥ 6 mois) et la prévalence de six mois d'un trouble mental et d'une multimorbidité (c'est à dire, ≥ 1 maladie physique et ≥ 1 trouble mental) chez les jeunes. Les associations entre la maladie physique et le trouble mental étaient quantifiées, notamment le nombre de maladies. Les objectifs secondaires examinaient les facteurs associés au trouble mental, après contrôle de la maladie physique.

**Méthodes:**

Les données proviennent 10 303 jeunes âgés de 4 à 17 ans de l'Étude sur la santé des enfants de l'Ontario (ESJO) de 2014. La maladie physique était mesurée à l'aide d'une liste d'affections chroniques développée par Statistique Canada. Les troubles mentaux étaient mesurés à l'aide des échelles de comportement émotionnel OCHS. Le Health Utility Index Mark III a évalué la santé fonctionnelle globale.

**Résultats:**

Les estimations pondérées de la prévalence ont montré que 550 090 (27,8 %) jeunes avaient une maladie physique, que 291 986 (14,8 %)avaient un trouble mental et que 108 435 (5,4 %) avaient une multimorbidité. La maladie physique n'était pas associée au trouble mental. Toutefois, les jeunes ayant deux maladies physiques, comparés à ceux n'en ayant aucune, avaient des probabilités accrues d'avoir un trouble mental (RC =1,5 [1,08, 2,5]), de l'humeur (RC =2,50 [1,39, 4,48]) et anxieux (RC =2,40 [1,33, 4,31]). Les scores moyens de santé fonctionnelle ont démontré une association dose-réponse dans toutes les catégories d'état de santé, les scores les plus élevés étant enregistrés chez les jeunes en bonne santé et les scores les plus faibles parmi les jeunes multi morbides (tous p < 0,05).

**Conclusions:**

Les maladies physiques chroniques et les troubles mentaux sont répandus chez les jeunes. Les jeunes atteints de deux maladies physiques sont plus susceptibles de souffrir de troubles mentaux. Des scores de santé fonctionnelle plus élevés protégeaient contre tous les troubles mentaux. Les interventions en santé mentale auprès des jeunes devraient promouvoir une bonne santé fonctionnelle globale.

## Introduction

Youth with chronic physical illnesses (i.e., those conditions that are expected to last at least 6 months), endure psychosocial and emotional challenges, predisposing them to an increased risk of mental disorder.^1–6^ Evidence suggests the risk of mental disorder is similar across different physical illnesses^1,7,8^ and increases with additional physical diagnoses^9,10^ and severity of functional limitations.^4,11^ Other biological and psychosocial risk factors of mental disorders include sex,^8,12,13^ age,^8,12,14^ lower socioeconomic status,^15^ and Indigenous status.^16^ The co-occurrence of at least 1 physical illness and at least 1 mental disorder (herein, *multimorbidity*), is common in youth.^1^ Prevalence of youth multimorbidity is estimated at 38% to 55% in Ontario-based clinical samples^1,17^ and 15% to 30% in Canadian and American epidemiological samples.^7,18^ Multimorbidity has negative impacts on quality of life,^9,18–20^ physical and mental health functioning,^18,21^ self-esteem,^20–23^ academic achievement,^24,25^ and suicidality.^26^ Because chronic physical illness and mental disorder are both chronic in nature, with at least 90% of youth with physical illness becoming adults with physical illness,^27^ and because the mental health impact of childhood physical illness persists across adolescence and into adulthood,^4,6^ an urgent public health response is needed to address the long-term consequences of youth multimorbidity.

Despite the chronicity of physical illness and mental disorder risk, there is a paucity of research that examines multimorbidity early in life. Evidence that does exist focuses on emerging adult, not youth, populations.^7,15,18,28^ Including children and adolescents in youth samples is critical for advancing the current state of knowledge about youth mental health and preventing mental disorders in this population. Unfortunately, estimates generated using non-standardized mental disorder diagnostic criteria^15^ make incidence and prevalence comparisons unreliable and assessments of burden difficult. Instead, using measures aligned with standardized diagnostic criteria (e.g., Diagnostic and Statistical Manual of Mental Disorders) is critical to public health prevention and surveillance efforts. Youth multimorbidity research often has outdated epidemiological samples,^18,28^ with contemporary samples needed to assess a broad spectrum of multimorbidity. Samples obtained from hospital registers^21^ limit generalizability to clinical populations, making population-level health recommendations difficult. Addressing these gaps in knowledge and methodology will produce evidence-based findings that can inform policy, prevention, and intervention efforts aimed at reducing mental disorders among youth with and without chronic physical illness.

The current study addresses these gaps in youth multimorbidity research by using contemporary epidemiological data that aligns with standard diagnostic criteria, from a sample of youth that is inclusive of children and adolescents. Data from the 2014 Ontario Child Health Study (OCHS) are used primarily to: (a) estimate the lifetime prevalence of chronic physical illness and 6-month prevalence of mental disorder and multimorbidity, in a sample of youth and (b) quantify associations between chronic physical illness and mental disorder, including the number of physical and mental morbidities. Secondary objectives include examining risk factors for mental disorders after controlling for the effect of chronic physical illness. Results will contribute to youth multimorbidity surveillance research and inform best practices for targeted youth mental health interventions.

## Methods

### Source

Analyses were conducted using the 2014 OCHS, a cross-sectional, epidemiological study implemented by Statistics Canada, that consisted of 10,802 youth from 6,587 households across 240 neighbourhoods.^29^ Multistage sampling, stratified by income, were used for sample selection, such that neighbourhoods were selected at the first stage, followed by census tracts and dissemination areas, and households were selected at the third stage.^29^ The target population included all private households in Ontario in which youth aged 4 to 17 years resided. Excluded were youth living in collective dwellings (e.g., youth in welfare and justice systems) as well as those living on Indigenous reserves (due to data sovereignty).^29^ The household response rate was 50.8%.^29,30^ In-person data collection occurred between October 2014 and September 2015, using computer-assisted interviews and self-completed paper questionnaires.^29^ The person most knowledgeable (PMK), 88.3% being mothers, was asked to complete surveys for up to 4 youths living in the household. The 2014 OCHS received ethical approval from the Hamilton Integrated Research Ethics Board (13–140).

### Measures

Responding to a list of chronic physical illnesses used in Statistics Canada surveys, the PMK reported if youth ever had physical illnesses diagnosed by a health professional that had lasted or were expected to last at least 6 months.^29^ Physical illnesses included: food or digestive allergies, respiratory allergies such as hay fever, any other allergies, asthma, bronchitis, cerebral palsy, diabetes, eczema, epilepsy, heart condition or disease, kidney condition or disease, and any other long-term conditions. A binary variable was created such that endorsement of at least 1 illness indicated the presence of chronic physical illness. Binary responses to a standard checklist are often used to examine chronic physical illness from a non-categorical approach as it is the consequences of physical illness, rather than a diagnostic label, that impacts mental health.^7,8^ An ordinal variable, based on the number of physical illnesses endorsed (0, 1, 2, or ≥3), assessed the cumulative impact of physical illnesses on mental disorders. The psychometric properties of this checklist are not reported by Statistics Canada; however, similar checklists show negligible misclassification bias.^31^

Mental disorder was measured using the PMK-reported OCHS Emotional Behavioural Scales (OCHS-EBS), a 52-item checklist, assessing symptoms of mental disorder in the previous 6 months.^30,32,33^ Based on DSM-5 criteria, the following mental disorders were assessed: major depression, generalized anxiety, separation anxiety, social phobia, conduct, oppositional defiant, and attention deficit hyperactivity disorder (ADHD). Each item was scored on a 3-point scale of (0) “never or not true”; (1) “sometimes or somewhat true”; and (2) “often or very true.” A total symptoms scale score was summed for all 7 disorders.^30^ Binary variables for any mental, mood (major depressive), anxiety (generalized, separation, and social phobia), behavioural (conduct and oppositional defiant), and neurodevelopmental disorders (ADHD) were created by establishing cut-points that resulted in closely matched disorder prevalence estimates reported in Georgiades, Duncan, Wang, et al. (2019). An ordinal variable was also created such that youth were grouped into having 0, 1, 2, or ≥3 mental disorders. The OCHS-EBS has an average test-retest reliability (based on к) of 0.67 and indicators of convergent validity (i.e., β coefficients) averaged 0.69.^33^

Overall functional health was measured using the Health Utilities Index Mark III (HUI). The HUI is a PMK-reported measure that assesses daily overall health and functioning across 8 health attributes: vision, hearing, speech, ambulation, dexterity, emotion, cognition, and pain.^29^ The HUI accounts for impairment, functional limitations, and reliance on aids and mechanical equipment (e.g., mobility and speech devices).^34^ A total score ranging from −0.34 (worse than death) to 1 (perfect health) was calculated, with 0 representing a death state.^34^ The HUI has been extensively validated across varying chronic illnesses and for youth population samples.^34,35^

Sociodemographic covariates were included in the analyses based on published evidence supporting their inclusion as risk factors for mental illness in youth with or without chronic physical illness and as potential confounders in the association between physical and mental illness.^8,12–16^ Covariates included age, sex, household income (low: <20th percentile of the sample median, mid: between low and high percentiles, and high: ≥80th percentile), and ethnicity (Black, Indigenous, other, Other Asian, South Asian, and White).

### Analysis

Descriptive and bivariable statistics were used to compare sociodemographic characteristics of youth based on health status (“healthy,” “physical illness only,” “mental disorder only,” and “multimorbid”). Cross-tabulations were used to calculate the lifetime prevalence of chronic physical illness and 6-month prevalence of mental disorders and multimorbidity (objective 1). Rao-Scott Chi-square tests compared sex-specific differences in prevalence rates of chronic physical illness and mental disorders. Prevalence estimates are presented as weighted percentages and frequencies. Using *t*-tests, overall functional health scores were compared among youth with and without physical illness and mental disorder. Logistic regression models were computed to quantify associations between chronic physical illness and mental disorder (objective 2a). Ordinal regression models quantified associations between chronic physical illness and a number of mental disorders (objective 2b). Sociodemographic and health covariates were simultaneously included in all regression models and adjusted results are reported with odds ratios (ORs) and 95% confidence intervals (CIs). Raw functional health scores were scaled by a factor of 10 to facilitate the interpretation of odds ratios.^36,37^ Due to the complex survey design and correlated responses amongst siblings from the same household, the balanced repeated replication variance estimation method was used with the generalized bootstrap weights and Fay adjustment to generate accurate standard errors. Weights were calculated by Statistics Canada to maintain representativeness with the Ontario youth population. Analyses were conducted in SAS 9.4.

### Missing Data

A total of 499 (4.6%) of youth had missing data in at least 1 covariate. Missing data for any mental disorder was associated with higher overall functional health scores (OR = 1.14 [1.04, 1.26]). Given the low level of missing data, complete case analysis was used,^38^ with a total sample of 6,242 households consisting of 10,303 youth, representing 1,976,819 Ontario youth.

## Results

### Sample Characteristics

The sex distribution of youth was nearly equal (1,018,635; 51.5% males), the mean age was 10.6 years (0.07), and the mean overall functional health score was 0.92 (0.002). Most youth came from 2-parent households (1,594,563; 80.8%), 1,236,916 (62.6%) were White, and 810,905 (41.0%) were immigrants. Almost half of PMKs (896,717; 46.1%) had at least a bachelor's degree and over half (1,131,886; 57.2%) of households reported a mid-level annual income. A dose–response association was observed for overall functional health scores, such that scores significantly decreased from “healthy” youth to “chronic physical illness only” to “mental disorder only” to “multimorbid” youth (*p *< 0.05 for all). As the number of physical illnesses and mental disorders cumulated, mean overall functional health scores decreased, with the lowest scores among youth with 3 or more physical illnesses (0.80, 0.01) and 3 or more mental disorders (0.60, 0.01). Detailed sample characteristics are shown in Table 1.

**Table 1.** Sample Characteristics.

		Morbidity status			
	Total	Healthy	CPI only	MD only	MM
Weighted *N*	1,976,819	1,243,177	441,655	183,552	108,435
% of total weighted sample (95% CI)	100	62.89 (61.19–64.58)	22.34 (20.84–23.83)	9.29 (8.33–10.23)	5.49 (4.64–6.32)
Age (mean, 95% CI)	10.59 (10.45–10.74)	10.27 (10.17–10.36)	11.21 (11.05–11.38)*	10.57 (10.32–10.82)	11.90 (11.56–12.23)
Health utility (mean, 95% CI)	0.92 (0.91–0.92)	0.96 (0.96–0.96)*	0.92 (0.91–0.93)*	0.78 (0.77–0.80)*	0.73 (0.71–0.76)*
Male	51.52 (49.80–53.26)	31.22 (29.62–32.82)	11.28 (10.16–12.40)	5.60 (4.83–6.36)*	3.42 (2.74–4.10)*
Ethnicity:					
Black	5.30 (4.61–5.99)	3.43 (2.90–3.97)	1.38 (0.98–1.79)	0.26 (0.10–0.41)	0.21 (0.08–0.36)
Indigenous	2.50 (1.99–3.01)	1.15 (0.86–1.44)	0.59 (0.28–0.90)	0.49 (0.26–0.72)	0.27 (0.09–0.44)
Other	6.09 (5.23–6.94)	4.07 (3.38–4.76)	1.34 (0.92–1.77)	0.47 (0.22–0.72)	0.20 (0–0.40)
Other Asian	11.64 (10.53–12.76)	8.14 (7.20–9.09)	2.47 (1.92–3.01)	0.78 (0.52–1.03)	0.24 (0.03–0.46)
South Asian	11.89 (10.71–13.07)	8.84 (7.82–9.87)	2.23 (1.68–2.79)	0.54 (0.23–0.84)	0.27 (0.08–0.47)
White	62.57 (60.89–64.25)	37.23 (35.57–38.90)	14.31 (13.05–15.58)	6.74 (5.93–7.54)	4.28 (3.53–5.02)
Lone parent household	19.19 (17.88–20.50)	11.55 (10.49–12.61)	3.92 (3.32–4.51)	2.12 (1.67–2.58)	1.59 (1.11–2.07)
Immigrant	41.02 (39.31–42.72)	28.66 (27.11–30.20)	8.74 (7.71–9.77)	2.49 (1.97–3.00)	1.12 (0.76–1.50)
Parent education:					
Did not complete secondary school	3.03 (2.54–3.52)	1.91 (1.52–2.30)	0.54 (0.33–0.74)	0.41 (0.21–0.62)	0.16 (0.06–0.26)
Secondary school completed	9.32 (8.39–10.24)	6.08 (5.30–6.84)	1.84 (1.43–2.24)	0.96 (0.65–1.28)	0.43 (0.25–0.62)
College, trade, or university below bachelor	41.54 (39.78–43.30)	24.79 (23.23–26.34)	9.80 (8.66–10.96)	4.05 (3.38–4.72)	2.89 (2.20–3.57)
Bachelor's degree or higher	46.1 (44.37–47.84)	30.09 (28.51–31.66)	10.19 (9.11–11.27)	3.81 (3.20–4.41)	2.01 (1.52–2.51)
Income:					
Low	20.70 (19.60–21.81)	12.83 (11.98–13.69)	4.38 (3.84–4.90)	2.24 (1.88–2.60)	1.25 (0.97–1.53)
Mid	57.26 (55.65–58.87)	35.98 (34.20–37.76)	12.88 (11.50–14.25)	5.14 (4.30–5.98)	3.26 (2.48–4.02)
High	22.03 (20.86–23.21)	14.07 (13.13–15.01)	5.09 (4.48–5.70)	1.90 (1.56–2.23)	0.97 (0.74–1.20)
Chronic physical illness:					
Any CPI	27.82 (26.22–2943)	∼	22.34 (20.85–23.83)	∼	5.49 (4.64–6.32)
1 CPI	19.47 (18.08–20.86)	∼	16.17 (14.87–17.47)	∼	3.30 (2.70–3.90)
2 CPIs	5.68 (4.80–6.56)	∼	4.17 (3.41–4.92)	∼	1.51 (1.03–1.99)
3≥ CPIs	2.67 (2.00–3.36)	∼	2.00 (1.44–2.57)	∼	0.67 (0.27–1.07)
Mental disorder:					
Any MD	14.77 (13.54–15.99)	∼	∼	9.28 (8.33–10.23)	5.49 (4.64–6.32)
1 MD	7.87 (6.96–8.78)	∼	∼	5.41 (4.68–6.16)	2.44 (1.89–3.00)
2 MDs	3.36 (2.71–4.00)	∼	∼	1.84 (1.44–2.25)	1.51 (1.00–2.02)
3 ≥MDs	3.54 (2.90–4.18)	∼	∼	2.01 (1.52–2.50)	1.52 (1.10–1.94)
Mood disorder	3.90 (3.20–4.59)	∼	∼	1.96 (1.50–2.42)	1.93 (1.40–2.47)
Anxiety disorder	6.33 (5.47–7.20)	∼	∼	3.72 (3.07–4.37)	2.61 (2.01–3.21)
Behaviour disorder	6.14 (5.31–6.96)	∼	∼	3.79 (3.18–4.40)	2.35 (1.78–2.92)
Neurodevelopment disorder (ADHD)	7.88 (7.02–8.73)	∼	∼	5.14 (4.45–5.84)	2.73 (2.21–3.26)

Data are frequency (95% CI) unless noted otherwise.

CPI = chronic physical illness; MD = mental disorder; MM = multimorbid; CI = confidence interval.

*Difference with all other health status groups is significant at *p* < 0.05.

### Prevalence of Chronic Physical Illness and Mental Disorders

From PMK reports, 62.9% (1,243,177) of youth were healthy (no physical illness or mental disorder), lifetime prevalence of chronic physical illness was 27.8% (550,090), 6-month prevalence of mental disorder was 14.8% (291,986), and 6-month multimorbidity was 5.4% (108,435). Specific mental disorder prevalence estimates were 3.9% (76,725) for mood, 6.3% (124,814) for anxiety, 6.1% (121,280) for behaviour, and 7.9% (155,261) for neurodevelopmental. Prevalences of each physical illness and mental disorder are shown in Supplementary Figures 1 and 2.

### Association between Chronic Physical Illness and Mental Disorder

Logistic regression models were computed to examine the association between having a chronic physical illness and mental disorder (Table 2). Across all adjusted models, chronic physical illness was not associated with mental disorder. Consistent across models, better overall functional health was associated with lower adjusted odds of mental disorder (OR range: 0.56–0.69). Age was associated with increased adjusted odds for mood (OR = 1.16 [1.10, 1.22]) and behaviour disorders (OR = 1.04 [1.01, 1.09]) and lower adjusted odds for neurodevelopmental disorder (OR = 0.91 [0.89, 0.95]). Female youth had lower adjusted odds for any mental (OR = 0.67 [0.54, 0.82]), behaviour (OR = 0.64 [0.49, 0.85]), and neurodevelopmental disorders (OR = 0.44 [0.34, 0.58]).

**Table 2.** Association of Chronic Physical Illness and Mental Disorder.

	Mental disorder				
	Any MD	Mood	Anxiety	Behaviour	Neurodevelopmental
	OR (95% CI)	OR (95% CI)	OR (95% CI)	OR (95% CI)	OR (95% CI)
CPI Present vs. not present (ref)	1.17 (0.94–1.45)	1.50 (0.95–2.34)	1.37 (0.96–1.96)	1.13 (0.81–1.57)	0.94 (0.71–1.25)
Demographics					
Age	0.99 (0.97–1.01)	**1.16** (**1.10–1.22)**	1.00 (0.97–1.04)	**1.04** (**1.01–1.09)**	**0.91** (**0.89–0.95)**
Sex: female vs. male (ref)	**0.67** (**0.54–0.82)**	0.82 (0.53–1.26)	1.17 (0.88–1.56)	**0.64** (**0.49–0.85)**	**0.44** (**0.34–0.58)**
Income:					
High vs. medium (ref)	0.95 (0.74–1.22)	0.68 (0.41–1.12)	0.77 (0.54–1.08)	0.71 (0.50–1.00)	1.10 (0.84–1.46)
Low vs. medium (ref)	1.12 (0.88–1.44)	1.02 (0.61–1.72)	1.01 (0.69–1.51)	1.10 (0.79–1.56)	1.24 (0.95–1.61)
Ethnicity:					
Black vs. White (ref)	**0.50** (**0.27–0.94)**	**0.07** (**0.03–0.14)**	**0.43** (**0.20–0.92)**	0.40 (0.16–1.00)	0.70 (0.36–1.38)
Indigenous vs. White (ref)	**1.84** (**1.12–3.02)**	**4.35** (**2.41–7.84)**	1.77 (0.78–4.02)	1.88 (0.90–3.88)	**2.19** (**1.33–3.60)**
Other vs. White (ref)	0.59 (0.35–1.00)	0.41 (0.17–1.03)	1.19 (0.58–2.46)	**0.42** (**0.23–0.74)**	0.59 (0.35–1.00)
Other Asian vs. White (ref)	**0.52** (**0.34–0.78)**	0.56 (0.23–1.33)	0.79 (0.43–1.44)	0.65 (0.41–1.01)	**0.49** (**0.28–0.83)**
South Asian vs. White (ref)	**0.39** (**0.25–0.61)**	**0.08** (**0.02–0.25)**	**0.39** (**0.20–0.76)**	**0.37** (**0.18–0.74)**	**0.40** (**0.19–0.84)**
Clinical functioning					
Health utility index	**0.56** (**0.51–0.60)**	**0.58** (**0.53–0.64)**	**0.64** (**0.60–0.70)**	**0.69** (**0.64–0.72)**	**0.57** (**0.52–0.60)**
*C*-Statistic	0.78	0.88	0.77	0.77	0.82

CPI = chronic physical illness; MD = mental disorder; OR = odds ratio; CI = confidence interval.

Results are reported as adjusted ORs and 95% CIs. Significant findings are shown in bold.

As compared to White youth, Black (OR = 0.50, [0.27, 0.94]), Other Asian (OR = 0.52 [0.34, 0.78]), and South Asian youth (OR = 0.39 [0.25, 0.61]) had lower adjusted odds of any mental disorder. Black (OR = 0.07 [0.03, 0.14) and South Asian youth (OR = 0.08 [0.02, 0.25]) had lower adjusted odds of mood disorder compared to White youth. For anxiety disorder, Black (OR = 0.43 [0.20, 0.92]) and South Asian youth (OR = 0.39 [0.20, 0.76]) had lower adjusted odds than White youth. For behaviour disorder, youth with “other ethnicity” (OR = 0.42 [0.23, 0.74]) and South Asian youth (OR = 0.37 [0.18, 0.74]) had lower adjusted odds compared to White youth. Youth categorized as Other Asian (OR = 0.49 [0.28, 0.83]) and South Asian (OR = 0.40 [0.19, 0.84]) had lower adjusted odds for neurodevelopmental disorder than White youth. Compared to White youth, Indigenous youth had higher adjusted odds for any mental (OR = 1.84 [1.12, 3.02]), mood (OR = 4.35 [2.41, 7.84]), and neurodevelopmental disorders (OR = 2.19 [1.33, 3.60]).

### Association of Number of Chronic Physical Illnesses and Mental Disorders

As shown in Table 3, having 2 chronic physical illnesses, as compared to no illness, was significantly associated with higher adjusted odds of having any mental (OR = 1.75 [1.08, 2.85]), mood (OR = 2.50 [1.39, 4.48]), and anxiety disorders (OR = 2.40 [1.33, 4.31]). There were no significant mental disorder outcomes for youth with 1 or 3 or more chronic physical illnesses. The effects of age, sex, income, ethnicity, and overall functional health on mental disorders were similar to those found for models of chronic physical illness and mental disorders.

**Table 3.** Association of Number of Chronic Physical Illnesses (3+) and Mental Disorder.

	Mental disorder				
	Any MD	Mood	Anxiety	Behaviour	Neurodevelopmental
	OR (95% CI)	OR (95% CI)	OR (95% CI)	OR (95% CI)	OR (95% CI)
Number of CPIs					
1 CPI vs. no CPI (ref)	1.06 (0.84–1.33)	1.31 (0.77–2.24)	1.20 (0.81–1.76)	1.16 (0.82–1.62)	0.93 (0.68–1.29)
2 CPIs vs. no CPI (ref)	**1.75** (**1.08–2.85)**	**2.50** (**1.39–4.48)**	**2.40** (**1.33–4.31)**	1.49 (0.82–2.69)	1.39 (0.82–2.32)
3 ≥ CPIs vs. no CPI (ref)	0.89 (0.33–2.34)	0.98 (0.27–3.52)	0.76 (0.14–4.08)	0.47 (0.12–1.78)	0.35 (0.10–1.26)
Demographics					
Age	0.99 (0.97–1.01)	**1.17** (**1.10–1.23)**	1.01 (0.97–1.04)	**1.05** (**1.01–1.09)**	**0.92** (**0.89–0.95)**
Sex: female vs. male (ref)	**0.67** (**0.54–0.82)**	0.83 (0.55–1.26)	1.18 (0.89–1.57)	**0.64** (**0.49–0.84)**	**0.44** (**0.34–0.57)**
Income:					
High vs. medium (ref)	0.94 (0.73–1.21)	0.68 (0.41–1.10)	0.77 (0.54–1.07)	0.71 (0.50–1.00)	1.10 (0.84–1.44)
Low vs. medium (ref)	1.13 (0.88–1.45)	1.04 (0.62–1.74)	1.03 (0.69–1.52)	1.11 (0.80–1.56)	1.25 (0.97–1.62)
Ethnicity:					
Black vs. White (ref)	**0.50** (**0.27–0.94)**	**0.07** (**0.03–0.14)**	**0.42** (**0.20–0.90)**	0.40 (0.16–1.00)	0.69 (0.35–1.37)
Indigenous vs. White (ref)	**1.74** (**1.04–2.89)**	**3.88** (**2.04–7.36)**	1.55 (0.67–3.61)	1.73 (0.81–3.70)	**2.00** (**1.21–3.31)**
Other vs. White (ref)	**0.59** (**0.35–0.99)**	0.41 (0.17–1.02)	1.17 (0.58–2.38)	**0.41** (**0.23–0.73)**	**0.58** (**0.34–0.98)**
Other Asian vs. White (ref)	**0.52** (**0.35–0.78)**	0.56 (0.23–1.33)	0.80 (0.43–1.46)	0.65 (0.41–1.01)	**0.49** (**0.29–0.83)**
South Asian vs. White(ref)	**0.39** (**0.25–0.60)**	**0.08** (**0.02–0.23)**	**0.38** (**0.19–0.76)**	**0.36** (**0.18–0.72)**	**0.39** (**0.18–0.82)**
Clinical functioning					
Health utility index	**0.56** (**0.51–0.60)**	**0.58** (**0.52–0.63)**	**0.64** (**0.59–0.69)**	**0.68** (**0.64–0.72)**	**0.56** (**0.52–0.60)**
*C*-Statistic	0.78	0.88	0.77	0.77	0.82

CPI = chronic physical illness; OR = odds ratio; CI = confidence interval.

Results are reported as adjusted ORs and 95% CIs. Significant findings are shown in bold.

### Number of Physical and Mental Morbidities

Ordinal regression models were computed to quantify the associations between the presence of chronic physical illness and the number of mental disorders (Table 4), and between the number of chronic physical illnesses and the number of mental disorders (Table 5). Results were robust across different analytic models, with the effects of chronic physical illness, age, sex, income, ethnicity, and health utility similar in Tables 2 and 3. As shown in Table 5, having 2 chronic physical illnesses increased the adjusted odds of the number of mental disorders (OR = 1.90 [1.20, 2.99]). There was no significant association with a number of mental disorders for youth with 1 or 3 or more chronic physical illnesses.

**Table 4.** Association of Any Chronic Physical Illness and Number of Mental Disorders (3+).

	Number of mental disorders
	OR (95% CI)
CPI	1.22 (0.99–1.51)
Present vs. not present (ref)	
Demographics	
Age	1.00 (0.97–1.01)
Sex: female vs. male (ref)	**0.67** (**0.54–0.81)**
Income:	
High vs. medium (ref)	0.91 (0.71–1.17)
Low vs. medium (ref)	1.08 (0.85–1.38)
Ethnicity:	
Black vs. White (ref)	**0.49** (**0.26–0.90)**
Indigenous vs. White (ref)	**1.93** (**1.21–3.08)**
Other vs. White (ref)	**0.59** (**0.34–0.99)**
Other Asian vs. White (ref)	**0.54** (**0.37–0.80)**
South Asian vs. White (ref)	**0.40** (**0.26–0.60)**
Clinical functioning	
Health utility index	**0.60** (**0.56–0.63)**
*C*-Statistic	0.77

CPI = chronic physical illness; OR = odds ratio; CI = confidence interval.

Results are reported as adjusted ORs and 95% CIs. Significant findings are shown in bold.

**Table 5.** Association of Number of Chronic Physical Illnesses and Number of Mental Disorders (3+).

	Number of mental disorders
	OR (95% CI)
Number of CPIs	
1 CPI vs. no CPI (ref)	1.09 (0.86–1.37)
2 CPIs vs. no CPI (ref)	**1.90** (**1.20–2.99)**
3 ≥ CPIs vs. no CPI (ref)	0.98 (0.42–2.29)
Demographics	
Age	1.00 (0.97–1.02)
Sex: female vs. male (ref)	**0.67** (**0.55–0.81)**
Income:	
High vs. medium (ref)	0.90 (0.71–1.16)
Low vs. medium (ref)	1.09 (0.86–1.38)
Ethnicity:	
Black vs. White (ref)	**0.49** (**0.26–0.90)**
Indigenous vs. White (ref)	**1.79** (**1.11–2.88)**
Other vs. White (ref)	**0.59** (**0.35–0.98)**
Other Asian vs. White (ref)	**0.55** (**0.37–0.81)**
South Asian vs. White (ref)	**0.39** (**0.26–0.60)**
Clinical functioning	
Health utility index	**0.60** (**0.56–0.63)**
*C*-Statistic	0.77

CPI = chronic physical illness; OR = odds ratio; CI = confidence interval.

Results are reported as adjusted ORs and 95% CIs. Significant findings are shown in bold.

## Discussion

### Summary of Findings

Using data from a large epidemiological study of Ontario youth, this study (a) estimated the lifetime prevalence of chronic physical illness and 6-month prevalence of mental disorder and multimorbidity and (b) quantified associations between chronic physical illness and mental disorder, including the number of physical–mental morbidities; and secondary objectives examined risk factors of mental disorder after controlling for physical illness. Regarding objective 1, over one-quarter of youth in the current sample reported chronic physical illness, aligning with contemporary epidemiological prevalence estimates.^39,40^ Compared to the seminal 1983 OCHS, chronic physical illness increased from 18.0%^41^ to 27.8%. The prevalence of youth screening positive for mental disorders (15%) was lower than estimates in epidemiological studies that report 1 in 5 youth living with mental disorders.^7,18,32^ In addition, youth multimorbidity decreased from 8.0%^41^ in the 1983 OCHS to 5.4% in the current study. Differences in mental disorder and multimorbidity estimates can be attributed to methodological differences in duration of illness (higher prevalence in longer study time periods), measurement of mental health (higher prevalence in studies that exclude impairment), structured diagnostic interviews vs. symptom checklist (prevalence differences based on type of disorder),^32^ number of mental disorders assessed, informant (higher rates in adolescent vs. parent reports),^32^ and respondent age (prevalence of different disorders fluctuate throughout development).^42^

Contrary to research suggesting a cumulative effect of a number of chronic physical illnesses on mental disorder risk,^9–11,43^ the current study found that youth with 2 chronic physical illnesses had increased odds of any, mood, and anxiety disorders as well as having increased odds of having a higher number of mental disorders (objective 2b). Notably, there were no significant associations with mental disorder outcomes for youth with 1 and 3 or more chronic physical illnesses. This finding may signal that youth in the general population living with 1 chronic physical illness may have relatively mild illness. With learned coping strategies, a single physical illness may be managed well by children and families, thus avoiding declines in mental health. Going from 1 to 2 chronic physical illnesses may result in more serious impairments, increasing the burden on youth and families, and thus increasing the odds of mental disorders. Youth with 3 or more chronic physical illnesses may have additional medical complexities, requiring routine follow-ups with health professionals—where in the context of holistic models of care—their mental health needs are adequately addressed.^44^ Alternatively, the mental health of medically complex youth may be overshadowed by their physical health needs. In addition, the mental health of medically complex youth may be difficult to assess due to substantial impairment or a lack of validated instruments for this specific population. These inferences are speculative; additional research to understand the nuances of physical–mental multimorbidity in youth is needed.

Overall functional health was a robust and consistent factor associated with mental disorders across different analytic models (secondary objectives). This finding supports the non-categorical approach to understanding youth multimorbidity.^7,8^ Rather than the presence or type of chronic physical illness, it is impairment and functional limitations (e.g., restrictions in dressing, walking, expressing thoughts independently)^35,45^ that conditions risk for mental disorder. As HUI score differences of 0.03 are clinically significant and the HUI responds to health changes over time^34^ it has been used to examine various treatment therapies in numerous cost-utility studies.^34^ Therapeutic interventions such as psychological counselling, physical and occupational therapy,^34^ medication adjustments,^35^ and use of aids (e.g., mobility and communication devices) can target functional limitations by addressing the barriers that reduce participation in developmentally appropriate activities. Future clinical research that incorporates the HUI as a measure of overall functional health, should examine modifiable functional limitations that are specific to physically and/or mentally ill youth. This study generated findings that were representative across the youth population and supported public health interventions targeting functional limitations, which aligns with the non-categorical approach to understanding multimorbidity in youth and young adult populations.^7,28,46^

The effects of age, sex, and ethnicity on mental disorders were generally consistent with current literature.^28,32,47,48^ Older age was associated with mood and behaviour disorders while younger age was associated with neurodevelopmental disorders. Females vs. males had lower odds of having behaviour and neurodevelopmental disorders. Whereas some studies report higher prevalences of internalizing disorders in females vs. males,^49,50^ the current study is consistent with other 2014 OCHS studies that did not find sex differences.^32,42^ Notably, Indigenous youth were disproportionately represented across mental disorders and multimorbidity. Compared to White youth, Indigenous youth were 4 times more likely to have a mood disorder and 2 times more likely to have a neurodevelopmental disorder. Given the current study excluded Indigenous youth living on reserve (about 37% of the Indigenous youth population)^29^ where mental disorder rates are approximately twice that in non-Indigenous communities,^51^ the current estimates are likely underestimated.^52^

### Implications

Findings support broad mental health screening across pediatric settings, regardless of diagnosis, for youth with a chronic physical and/or mental disorder. Vigilant screening efforts are needed for youth with 2 physical illnesses. Strategies should involve routine monitoring of physical and mental health, impairment, and functioning. Interventions should target the prevention of functional health declines, which would promote favourable health outcomes over the life course. These strategies would address continued recommendations to integrate physical and mental health care.^8,53^ Given the higher odds of mental disorders among Indigenous youth, integrated health care and mental health interventions must be culturally appropriate. Future research should examine the nuances in the association between the number of physical illnesses and mental disorder outcomes, including potential mediating and moderating effects. Future clinical research should examine interventions to prevent functional health declines and the impact that income, age, sex, and ethnicity may have on accessing therapeutic interventions.

### Strengths and Limitations

The current study contributes to youth multimorbidity research, an emerging field of study. As a large-population-based study, findings can be broadly applied to the spectrum of youth, inclusive of children and adolescents. There are significant implications for health policy, supporting youth with and without chronic physical illness. Modifiable impairments and functional limitations can be targeted to improve mental health outcomes in youth with physical and/or mental illnesses. Potential biases associated with cross-sectional data (e.g., recall and misclassification biases) are minimized by using health professional-diagnosed conditions and standardized mental health measures that align with DSM-5 criteria.

There are limitations warranting consideration when interpreting findings from this study. First, data were collected before the COVID-19 pandemic; thus, the current burden of chronic physical illness, mental disorder, and multimorbidity may be underestimated.^17^ Second, because the parent version of the OCHS-EBS was used, the reported prevalence of internalizing mental disorders and thus multimorbidity may be underestimated.^32,54,55^ Future research should include youth self-reports and where possible, other informants (e.g., teacher, health professional). Third, prevalence estimates may be under or overestimated due to racial and cultural biases in assessments and reporting of mental disorders. For instance, assessments may overestimate externalizing disorders for Black youth while estimates for Asian youth may be underreported.^47,48^ Last, the 2014 OCHS is cross-sectional and causation cannot be inferred.

## Conclusion

Chronic physical illness and mental disorders are prevalent in youth. While the presence of chronic physical illness was not associated with mental disorders, the number of chronic physical illnesses was important in examining mental health outcomes. Youth with 2 chronic physical illnesses have increased odds of mental disorders. Indigenous youth were consistently more likely to report mental disorders, providing evidence for the importance of culturally sensitive health care. Even after adjusting for relevant health factors, better overall functional health was robustly associated with better mental health outcomes across various analytical models. A dose–response was observed, such that overall daily health functioning scores significantly declined from “physical illness only” to “mental disorder only” to “multimorbidity.” Future clinical research should examine targeted interventions aimed at preventing functional health declines, supporting a mental health research strategy for youth with and without chronic physical illness.

## Supplemental Material

sj-docx-1-cpa-10.1177_07067437241271713 - Supplemental material for An Epidemiological Study of Physical–Mental Multimorbidity in Youth: Une étude épidémiologique de la morbidité physique-mentale chez les jeunes Supplemental material, sj-docx-1-cpa-10.1177_07067437241271713 for An Epidemiological Study of Physical–Mental Multimorbidity in Youth: Une étude épidémiologique de la morbidité physique-mentale chez les jeunes by Shannon V. Reaume, Joel A. Dubin, Christopher Perlman and Mark A. Ferro in The Canadian Journal of Psychiatry

sj-docx-2-cpa-10.1177_07067437241271713 - Supplemental material for An Epidemiological Study of Physical–Mental Multimorbidity in Youth: Une étude épidémiologique de la morbidité physique-mentale chez les jeunes Supplemental material, sj-docx-2-cpa-10.1177_07067437241271713 for An Epidemiological Study of Physical–Mental Multimorbidity in Youth: Une étude épidémiologique de la morbidité physique-mentale chez les jeunes by Shannon V. Reaume, Joel A. Dubin, Christopher Perlman and Mark A. Ferro in The Canadian Journal of Psychiatry
