# Supplementary material for: An Epidemiological Study of Physical–Mental Multimorbidity in Youth: Une étude épidémiologique de la morbidité physique-mentale chez les jeunes
Source: Can J Psychiatry. 2024 Aug 16;69(10):749–58. doi: 10.1177/07067437241271713 (PMC11485671; doi:10.1177/07067437241271713)
Supplement: sj-docx-2-cpa-10.1177_07067437241271713 - Supplemental material for An Epidemiological Study of Physical–Mental Multimorbidity in Youth: Une étude épidémiologique de la morbidité physique-mentale chez les jeunes [file sj-docx-2-cpa-10.1177_07067437241271713.docx]

**Supplementary Figure 1.**

**Prevalence of Chronic Physical Illnesses.**
